# Impact of COVID-19 Mitigation Measures on Mosquito-Borne Diseases in 2020 in Queensland, Australia

**DOI:** 10.3390/v13061150

**Published:** 2021-06-16

**Authors:** Cassie C. Jansen, Jonathan M. Darbro, Frances A. Birrell, Martin A. Shivas, Andrew F. van den Hurk

**Affiliations:** 1 Communicable Diseases Branch, Department of Health, Queensland Government, Herston, Brisbane, QLD 4006, Australia; frances.birrell@health.qld.gov.au; 2 Metro North Public Health Unit, Queensland Health, Windsor, Brisbane, QLD 4030, Australia; jonathan.darbro@health.qld.gov.au; 3 Field Services, Brisbane City Council, Eagle Farm, Brisbane, QLD 4009, Australia; martin.shivas@brisbane.qld.gov.au; 4 Public Health Virology, Forensic and Scientific Services, Department of Health, Queensland Government, Coopers Plains, Brisbane, QLD 4108, Australia

**Keywords:** COVID-19, mosquito, arbovirus, Ross River virus, transmission, Australia

## Abstract

We describe the impact of COVID-19 mitigation measures on mosquito-borne diseases in Queensland, Australia, during the first half of 2020. Implementation of restrictions coincided with an atypical late season outbreak of Ross River virus (RRV) characterized by a peak in notifications in April (1173) and May (955) which were greater than 3-fold the mean observed for the previous four years. We propose that limitations on human movement likely resulted in the majority of RRV infections being acquired at or near the place of residence, and that an increase in outdoor activities, such as gardening and bushwalking in the local household vicinity, increased risk of exposure to RRV-infected mosquitoes. In contrast, the precipitous decline in international passenger flights led to a reduction in the number of imported dengue and malaria cases of over 70% and 60%, respectively, compared with the previous five years. This substantial reduction in flights also reduced a risk pathway for importation of exotic mosquitoes, but the risk posed by importation via sea cargo was not affected. Overall, the emergence of COVID-19 has had a varied impact on mosquito-borne disease epidemiology in Queensland, but the need for mosquito surveillance and control, together with encouragement of personal protective measures, remains unchanged.

## 1. Introduction

The coronavirus infectious disease 19 (COVID-19) pandemic caused by severe acute respiratory syndrome coronavirus 2 (SARS-CoV-2) is an unprecedented event that has significantly impacted the social, political and economic infrastructure of many countries [1]. The first case of COVID-19 in Australia was diagnosed in January 2020 in a traveller from China [2]. The number of cases and deaths attributed to COVID-19 notified in Australia, to 6 May 2021, was 29,865 and 910, respectively [3].

The first COVID-19 infection wave in Australia occurred in the first half of 2020 and affected all states and territories, peaking in March before waning in April. This first wave was primarily associated with overseas-acquired infection and subsequent clusters of community transmission. Starting in June 2020, a second wave of transmission occurred, largely localized to the state of Victoria, and was mostly associated with community acquired infections. In response to the emergence of COVID-19 in Australia, numerous measures were implemented to prevent and suppress transmission including restricting entry by non-citizens and non-residents, mandatory quarantine for international travellers, self-isolation for diagnosed COVID-19 cases, limitation of movement outside residents’ homes and strict physical distancing requirements [4]. These measures had widespread and varied impact across education, employment, health, trade, tourism, sport and recreation, religious practice and regular social community practices and gatherings.

Essential strategies employed to limit COVID-19 transmission have likely had unforeseen and unintended impacts on the epidemiology and management of other diseases, including those caused by mosquito-borne pathogens [5,6]. Redeployment of specialist resources and personnel away from mosquito and mosquito-borne disease prevention and control programs, the effect of sustained lockdowns and restricted social movement and travel on virus transmission dynamics, and restricted access to productive mosquito habitats by mosquito control personnel were considered some of the likely consequences of COVID-19 prevention and response initiatives [7,8].

Australia possesses a complex pattern of mosquito-borne pathogen transmission [9] due to its climate, location, connectivity with hyperendemic countries, unique vertebrate fauna and diversity of mosquito species. We describe the status of mosquito-borne pathogens in the state of Queensland, Australia, in the first six months of 2020 when emergency measures were implemented to limit the impact of COVID-19.

## 2. Unseasonal RRV Transmission

Of the 75 arboviruses that have been identified in Australia, Ross River virus (RRV) infections account for the largest number of human mosquito-borne disease notifications. For the reporting period 1 July 2014 to 30 June 2019, a five-year annual mean of 5535 cases were notified nationally [10] with a large outbreak observed in 2014–2015 [11]. Infection with RRV causes a spectrum of disease ranging from asymptomatic infection to debilitating polyarthritis and can include fever, maculopapular rash, fatigue, myalgia, lethargy, and/or headache [12,13]. The virus exists in a complex zoonotic transmission cycle between a variety of vertebrate hosts and mosquito species [14]. Whilst human cases of RRV infection are notified to health authorities throughout all months of the year, outbreaks of RRV periodically occur and are linked to the widespread availability of larval habitats of mosquito vectors caused by above average rainfall and/or king tides (in coastal areas) [15]. Historically, RRV cases in Queensland begin to rise with the onset of rain and elevated temperatures in December before peaking in February–March [11,16].

**Figure 1:**
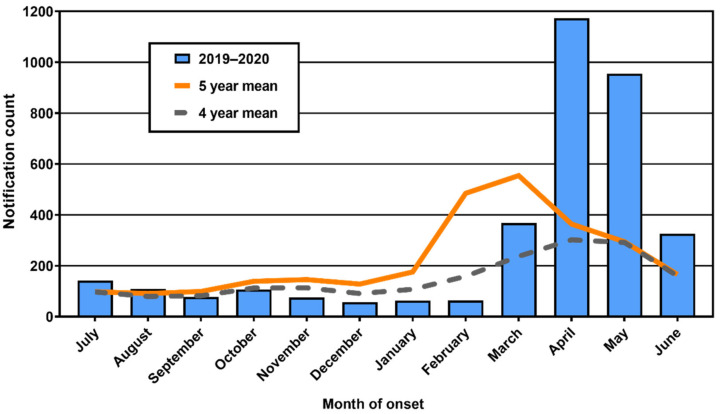
Ross River virus infection notifications in Queensland by month for the 2019–2020 reporting year. Monthly means for the previous 4 and 5 reporting years are provided for comparison. Note that 5-year mean includes notifications comprising a large outbreak in 2014–2015.

In Queensland, mean monthly case counts of RRV for the 2019–2020 Spring–Summer season were comparable with or lower than historical means in the last half of 2019 but showed a late and marked increase in numbers of cases from March 2020 (Figure 1). During January to June 2020, 2949 cases of RRV infection were reported to Queensland Health. The 2020 count was 2.3-fold the mean (1256) for the same period during the previous four years. The highest notification counts were reported in April and May, with 1173 and 955, respectively, which were 3.9- and 3.3-fold the mean count for these months over the previous four years. Cases declined in June, but notifications for this month were still twice the historical mean.

**Figure 2:**
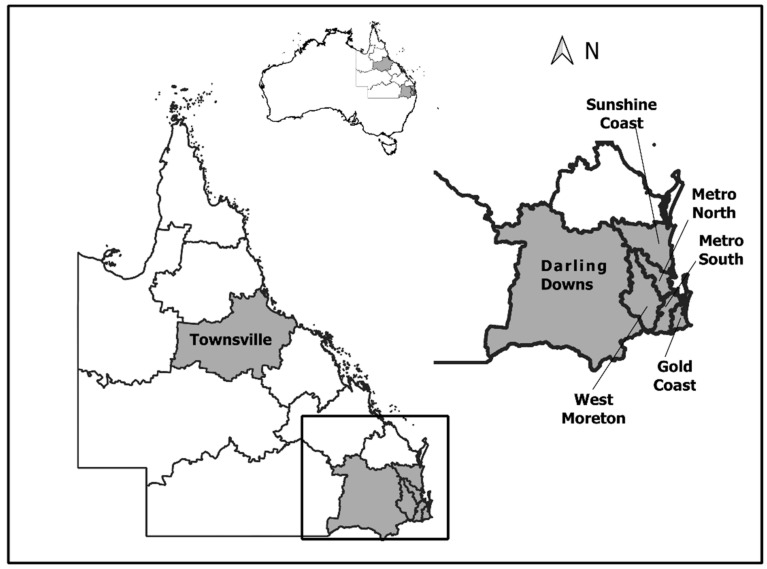
Queensland Hospital and Health Service (HHS) areas that reported higher than expected Ross River virus notifications for the period January to April 2020.

This pattern of increased notifications was not consistent across the state of Queensland, with seven of the 15 Queensland Hospital and Health Service (HHS) areas reporting higher than the mean late season RRV notifications (Figure 2). Six of these HHS areas were in the southeast region (Sunshine Coast, Metro North, Metro South, West Moreton, Darling Downs and Gold Coast), in addition to the northern HHS area of Townsville.

The temporal pattern of RRV notifications in early 2020 was markedly different to that observed in other years and was inconsistent with observations of mosquito vector densities. Notably, the numbers of notifications of human RRV occurred much later (with a peak occurring in April and higher than usual numbers of notifications persisting in May) than the typical seasonal peak, presumably reflecting very late epizootic transmission and human exposure risk. Whilst there is considerable heterogeneity in rainfall across Queensland, both high rainfall and king tides were reported in Southeast Queensland in December 2019 and the first three months of 2020 [17]. In Brisbane, this led to relatively high observed numbers of *Aedes vigilax* and *Culex annulirostris*, two abundant and important vectors of RRV, throughout this period (Figure S1). However, with the onset of dry conditions from April, mosquito population densities rapidly decreased and generally reached low levels by May and June. Thus, there appeared to be a temporal lag between high densities of common mosquitoes driven by rain and tidal events (Figure S1), and the increased notifications of RRV infection. Explanations for this lag include the possibility that species other than *Aedes vigilax* and *Culex annulirostris* (which both dominate collections in mosquito surveillance traps) may have been responsible for late season transmission to humans. Alternatively, the high mosquito numbers observed earlier in 2020 may have facilitated widespread epizootic virus amplification within the reservoir vertebrate population ensuring that even when mosquito numbers declined to lower levels, the high prevalence of virus in vertebrate reservoirs was sufficient to trigger virus spill over into the human population.

Peak numbers of RRV notifications coincided with Level 3 COVID-19 restrictions, which were implemented in Queensland from late March 2020 [18]. These restrictions included encouraging residents to stay at home where possible, limiting movement to essential travel to a maximum of 50 km from place of residence, reducing the number of attendees to indoor and outdoor gatherings to prescribed numbers, and restricting recreational activities, such as camping and fishing. Furthermore, to assist in social/physical distancing, employees were encouraged to work from home wherever possible, most primary and secondary students transitioned to home schooling, and virtually all indoor and outdoor activities were cancelled or substantially restricted in scope, limiting the number of persons allowed to gather.

As RRV is a notifiable condition in Australia, details of cases are recorded on the notifiable conditions system (NOCS [19]), including the residential address of each case. Typically, there is no assurance that place of residence truly reflects location of RRV acquisition. Given the dramatic reduction in human gatherings, long-distance travel and short-range social movements during this period, it can be postulated that most RRV infections were acquired at or close to the place of residence of a case, rather than being associated with mosquito exposure whilst undertaking recreational activities that are typically located away from the place of residence, such as fishing or camping. Indeed, the latter activity has previously been linked to increased risk of acquiring RRV infection [20].

Whilst the distance that residents could travel from their homes was limited, reports indicate that people may have changed their exercise habits to encompass outdoor activities, such as gardening and bushwalking in the immediate vicinity of the home [21]. An increase in the frequency of these local outdoor activities could potentially have exposed residents to mosquitoes if populations persisted in focal locations close to their homes. For instance, there was a distinct cluster of cases of RRV infection recorded proximal to a large conservation reserve in northern Brisbane where the numbers of people exercising had potentially increased. Thus, it seems that a change in the movement patterns of residents likely caused a change in the spatial pattern of exposure risk and that the spatial distribution of RRV notifications in 2020 may more accurately reflect the spatial distribution of vectors and hosts than in other years. Interestingly, in Sydney, the state capital of New South Wales, increased RRV notification rates were linked to changes in physical activity due to the COVID-19 lockdown and elevated mosquito populations caused by higher than average rainfall [22]. Similar changes in human behavior in response to COVID-19 restrictions were linked to increased cases of tick-borne encephalitis virus infection in some locations in Germany [23]. Regardless of the impact of COVID-19, further eco-epidemiological studies are clearly required to define the transmission dynamics of RRV in foci such as the nature reserve described above, where a range of both potential vertebrate hosts and mosquito larval habitats occur.

## 3. Importation of Exotic Pathogens by Overseas Travellers

Whilst dengue viruses (DENVs) and malaria are not endemic, their importation via infected travellers is a constant threat to Australia, particularly in north Queensland which has a history of local DENV and malaria transmission precipitated by viremic or parasitaemic arriving travellers [24,25]. The vulnerability of this region is attributable to the presence of the two key dengue virus vectors, *Aedes aegypti* and *Aedes albopictus*, and mosquito members of the *Anopheles farauti* species complex, the primary potential vectors of malaria parasites, as well as a high influx of travellers from dengue and malaria endemic locations in the region.

As part of the COVID-19 response, the Australian Government implemented strict border entry restrictions in early February 2020, commencing with travellers from China. Gradually, travel from additional countries was likewise restricted, before Australia’s borders were closed to non-citizens and non-residents, and mandatory quarantine of 14 days upon arrival was imposed on all international arrivals [4]. This resulted in a precipitous decline of 98% in the number of international visitors and returned residents when compared with the same period in 2019 [26].

During the first half of 2020, Queensland Health was notified of 50 cases of overseas-acquired dengue, compared with a mean of 181 for the same period during the preceding 5 years. Similarly, there were 96 notifications of malaria during January to June 2020, compared with the 5-year mean of 258. Clearly, international travel restrictions implemented by the Australian Government to limit COVID-19 indirectly contributed to the observed declines in imported mosquito-borne disease cases and a concomitant decrease in the risk posed by these pathogens.

## 4. Exotic and Invasive Mosquitoes

Although *Aedes aegypti* is present in much of north Queensland, and in some locations in central and southern Queensland, and *Aedes albopictus* is prevalent throughout the Torres Strait, the remaining regions of Australia are considered free from these species [27,28]. In these uninfested locations, there is ongoing concern that these species could become established and render them receptive to autochthonous transmission of viruses, such as DENVs, chikungunya and Zika (ZIKV), should they be imported [29]. To reduce the likelihood of invasion and/or establishment by *Aedes aegypti* and/or *Aedes albopictus*, surveillance and control programs are conducted in vulnerable locations by federal, state and local government authorities.

Prevention and mitigation of the risk of importation of exotic invasive mosquitoes via international travel has been at the forefront of public health and biosecurity efforts in recent years. On the mainland, the Federal Department of Agriculture, Water and Environment undertakes surveillance for exotic mosquitoes on incoming cargo and vessels, and within 400 m of International First Points of Entry [30]. This surveillance is supplemented by monitoring performed by state and local government personnel. Both *Aedes aegypti* and *Aedes albopictus* are regularly intercepted in imported cargo, such as oversized tyres and other break-bulk items, and in association with vessels and aircraft arriving from infested ports of origin [31]. In recent years, exotic mosquitoes have been intercepted at both air- and seaports, associated predominantly with passenger and cargo traffic, respectively.

During the period when COVID-19 restrictions were imposed, the number of incoming passenger flights decreased considerably, thus potentially limiting this incursion pathway. Indeed, there were no interceptions of exotic mosquitoes associated with passenger terminal arrivals during this period, although it is unknown if this can be attributed to changed air travel patterns alone. In contrast to air passenger flights, when COVID-19 restrictions were implemented from April to June 2020, there was only a small decline in the tonnage of cargo imported into the Port of Brisbane when compared to the mean of the previous 5 years for these months (Port of Brisbane Pty Ltd., Brisbane, Australia, unpublished data). Thus, this pathway was not overtly impacted by COVID-19, although there were no interceptions of exotic mosquitoes at the seaport reported during this period. It is notable, however, that the re-deployment of public health staff to the COVID-19 response, in conjunction with a suspension of some site visits to limit risk of potential exposure of staff to SARS-CoV-2 at passenger and freight terminals, led to the downscaling or suspension of some exotic mosquito surveillance programs in southeast Queensland.

Another invasive mosquito incursion pathway of concern to the Australian mainland is via the Torres Strait, where *Aedes albopictus* has been established since at least 2004 [32]. A comprehensive suppression strategy to minimize risk to the mainland has, to date, contained *Aedes albopictus* populations on Thursday and Horn Islands, which are the main transport hubs to the mainland [33]. To protect vulnerable remote communities from COVID-19, restrictions on travel to the Torres Strait were implemented in March 2020 [18]. This limited the ability of mosquito control personnel (based on the mainland) to travel to the Torres Strait and undertake routine surveillance and insecticide treatments targeting *Aedes albopictus*. Fortunately, in this instance there was no observed increase in *Aedes albopictus* populations once surveillance and control activities resumed after six weeks, possibly because of the residual effects of pyrethroid insecticide applied immediately prior to access being restricted (O. Muzari, unpublished data). This was different to a previous year when inclement weather forced the suspension of insecticide applications early in the year and *Aedes albopictus* numbers increased dramatically. This highlights the unintended impact that reduced travel, in this case due to COVID-19, can have on existing and critical mosquito suppression programs.

## 5. Impact on Workforce

Federal, state and local government agencies, with input from research institutions, are charged with understanding and mitigating the impacts of mosquito-borne diseases in Australia. Diverse disciplines work together to address these risks and these efforts include medical entomologists, mosquito-control personnel, medical and public health officers, epidemiologists, diagnostic scientists, veterinarians, biosecurity officers and environmental health officers. In Australia, mosquito-borne disease surveillance and control programs have been affected by competing workplace priorities, social/physical distancing measures, constrained resources and reallocation of funding, all associated with the emergence of COVID-19 and organizations working under business continuity conditions. Many public health personnel were, understandably, redeployed to COVID-19 response efforts across Australia. Whilst their involvement in COVID-19 mitigation and response activities have been diverse, these constraints have variably suspended many non-essential components of mosquito-borne disease prevention and control programs. Nevertheless, most routine state-wide surveillance programs have continued, albeit with reduced scope in some instances. Local government mosquito control efforts have largely been maintained in most instances, although workflows had to be modified to incorporate COVID-19 safe working practices, including interactions with external contractors. This impact is similar to that described in Florida, where mosquito control programs mostly continued to function during lockdowns, although arbovirus surveillance systems were impacted because diagnostic capability was diverted to SARS-CoV-2 testing [34]. Whilst these impacts have been unavoidable and essential, the downstream effects on operational mosquito management should not be dismissed.

## 6. Communicating Evidence That Mosquitoes Are Unlikely to Be Vectors of SARS-CoV-2

Health crises generate a need to communicate trusted information. When COVID-19 emerged, medical entomologists and arbovirologists fielded questions from the public regarding potential transmission of SARS-CoV-2 by mosquitoes. The response was that unlike true arboviruses, such as the DENVs, RRV and ZIKV, it was highly unlikely that SARS-CoV-2 would be able to infect or be transmitted by mosquitoes. As experimental evidence supporting this advice accumulated in the scientific literature [35,36], it greatly assisted community messaging. Whilst there remains no scientific evidence to support the suggestion that mosquitoes may be vectors of SARS-CoV-2, public concern and media coverage associated with this unfounded concern has undoubtedly distracted from other health messaging regarding actual mosquito-borne disease risk.

## 7. Conclusions

The COVID-19 pandemic has not only had a catastrophic disease impact but has, in many cases, applied constraints on health authorities to respond to other public health needs, including mosquito-borne diseases. Queensland recorded relatively few total cases and limited local transmission of SARS-CoV-2 in comparison with most of the rest of the world. Nevertheless, the essential implementation of social/physical distancing measures and restricted travel to protect the population also likely affected the observed epidemiology, surveillance and control of mosquito-borne pathogens in the state. Whilst undoubtedly causing a change to the spatial pattern of exposure risk of residents acquiring endemic mosquito-borne diseases, movement and travel restrictions perhaps unsurprisingly reduced the risk of local outbreaks of exotic pathogens. Redeployment of public health and mosquito management expertise and capacity arguably reduced the effort available for mosquito and mosquito-borne disease surveillance and control. Importantly, this experience highlighted that mosquito avoidance measures, such as eschewing activities when mosquitoes are most active, applying personal repellents, using spatial repellents, and wearing long-sleeved clothing are just as important in the uncertain times caused by COVID-19 as they have always been.

## Data Availability

Authorization for the use of human disease data from the Queensland Notifiable Conditions Register was obtained from the Communicable Diseases Branch, Department of Health, Queensland Government.

## Supplementary Materials

The following are available online at https://www.mdpi.com/article/10.3390/v13061150/s1: Figure S1: Seasonal abundance of the six historically most abundant mosquito species at an indicative surveillance site, northern Brisbane from July 2012 to June 2020. Note the different scales for the y-axis. The mosquito referred to as *Verrallina* Marks sp. No.32 was recognized as a separate species by the late E.N. Marks but has not been formally named.
