# Supplementary figures and images for: Impact of COVID-19 Mitigation Measures on Mosquito-Borne Diseases in 2020 in Queensland, Australia
Source: Viruses. 2021 Jun 16;13(6):1150. doi: 10.3390/v13061150 (PMC8235246; doi:10.3390/v13061150)

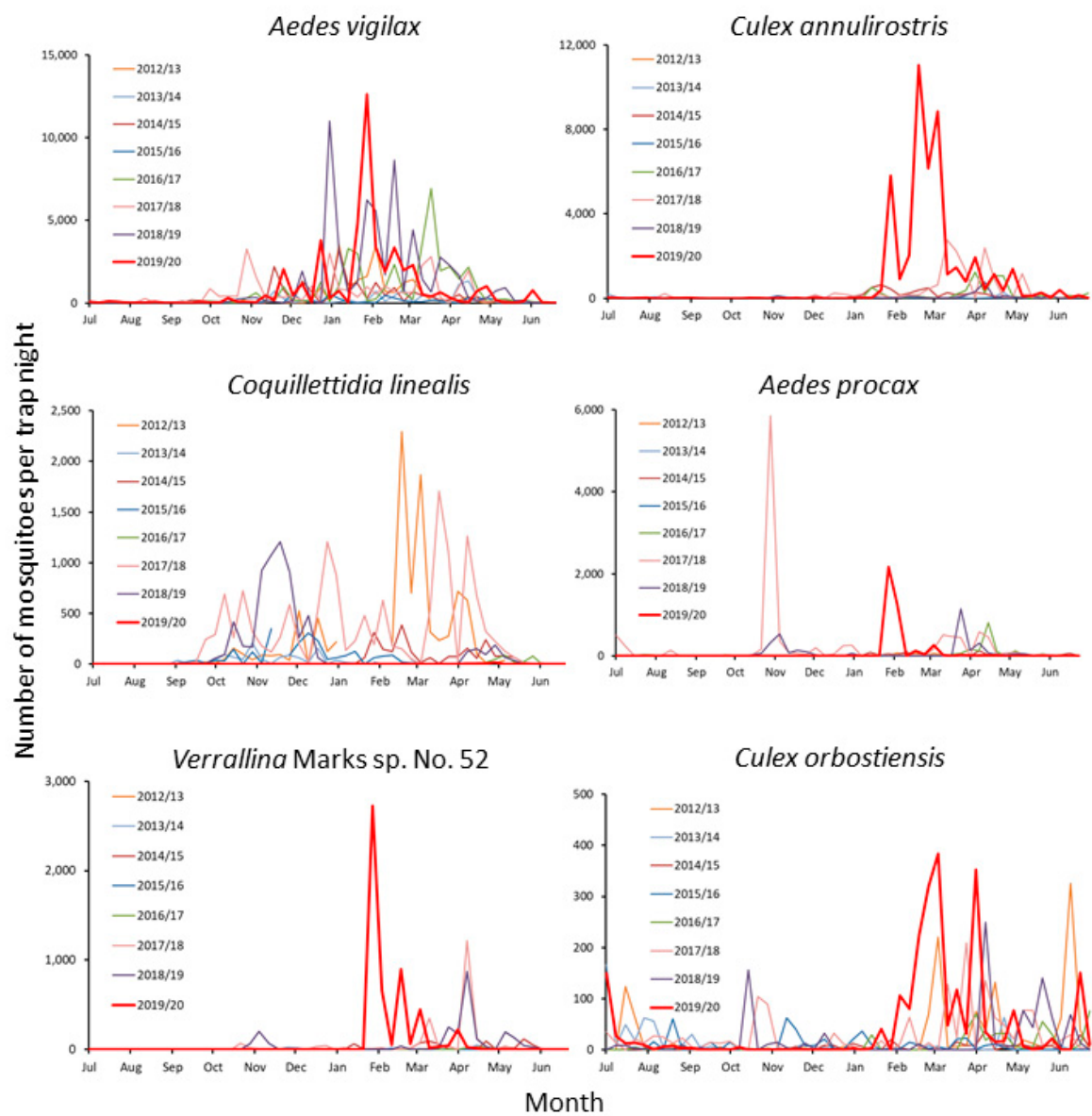

Supplement: Supplementary file 1 [file viruses-13-01150-s001.zip › viruses-1254770-supplementary.pdf]
